# Changes in total cholesterol level and cardiovascular disease risk among type 2 diabetes patients

**DOI:** 10.1038/s41598-023-33743-6

**Published:** 2023-05-23

**Authors:** Jaewon Khil, Sung Min Kim, Jooyoung Chang, Seulggie Choi, Gyeongsil Lee, Joung Sik Son, Sang Min Park, NaNa Keum

**Affiliations:** 1 Department of Food Science and Biotechnology, Dongguk University Graduate School, Seoul, Korea; 2 Department of Nutrition, Harvard T.H. Chan School of Public Health, Boston, MA USA; 3 Department of Biomedical Sciences, Seoul National University Graduate School, Seoul, Korea; 4 Department of Internal Medicine, Seoul National University Hospital, Seoul, Korea; 5 KS Health Link Inst. and Life Clinic, Seoul, Korea; 6 Department of Internal Medicine, Hallym University Sacred Heart Hospital, Anyang, Korea; 7 Department of Family Medicine, Seoul National University Hospital, Seoul, Korea

**Keywords:** Public health, Preventive medicine

## Abstract

Despite many diabetic patients having hypercholesterolemia, the association of total cholesterol (TC) levels with CVD risk in type 2 diabetes (T2D) patients is unclear. Diagnosis of type 2 diabetes often leads to changes in total cholesterol (TC) levels. Thus, we examined whether changes in TC levels from pre- to post-diagnosis of T2D were associated with CVD risk. From the National Health Insurance Service Cohort, 23,821 individuals diagnosed with T2D from 2003 to 2012 were followed-up for non-fatal CVD incidence through 2015. Two measurements of TC, 2 years before and after T2D diagnosis, were classified into 3 levels (low, middle, high) to define changes in cholesterol levels. Cox proportional hazards regression was performed to estimate adjusted hazards ratios (aHRs) and 95% confidence intervals (CIs) for the associations between changes in cholesterol levels and CVD risk. Subgroup analyses were performed by use of lipid-lowering drugs. Compared with low–low, aHR of CVD was 1.31 [1.10–1.56] for low–middle and 1.80 [1.15–2.83] for low–high. Compared with middle–middle, aHR of CVD was 1.10 [0.92–1.31] for middle–high but 0.83 [0.73–0.94] for middle–low. Compared with high–high, aHR of CVD was 0.68 [0.56–0.83] for high–middle and 0.65 [0.49–0.86] for high–low. The associations were observed regardless of use of lipid-lowering drugs. For diabetic patients, management of TC levels may be important to lower CVD risk.

## Introduction

Cardiovascular disease (CVD) is the leading cause of death globally^1,2^. The World Health Organization (WHO) predicts that more than 23 million people will die of CVD by 2030^2^. A major gateway disease to CVD is type 2 diabetes (T2D)^3^. In a meta-analysis of 30 cohort studies, diabetic patients had 1.52 times increased risk of CHD and 1.23 times increased risk of stroke compared with non-diabetic patients^4^. With the worldwide prevalence of T2D reaching 9.3% and expected to increase to 10.2% by 2030^5^, efforts prevent CVD among diabetic patients are of public health importance.

One strong risk factor for CVD in a healthy population is hypercholesterolemia^6,7^, and its adverse effect on CVD might be more evident among individuals with underlying metabolic disease like T2D. In a nationwide cohort study conducted in Korea, among patients with T2D, levels of circulating low-density lipoprotein cholesterol (LDL-C) at which myocardial infarction (MI) risk started to increase were ≥ 130 mg/dL for statin non-users and ≥ 70 mg/dL for statin users^8^. These thresholds are lower than 160 mg/dL, which defines high LDL-C in the general population. Thus, diabetic patients may be more vulnerable to the adverse effect of hypercholesterolemia on CVD risk of T2D. However, diagnosis of T2D often leads to positive lifestyle modification, which helps lower the risk of hypercholesterolemia and CVD. The net effect of these divergent factors on cholesterol levels is captured in changes in cholesterol levels around the diagnosis of T2D. Despite that an estimated 34.9% of diabetic patients also have hypercholesterolemia^9^, evidence on the relationship between cholesterol levels and CVD risk among diabetic patients are scarce. Therefore, we investigated how changes in total cholesterol (TC) levels from pre- to post-diagnosis of T2D are associated with subsequent CVD risk, accounting for the use of lipid-lowering drugs including statin, fibrate, and ezetimibe.

## Results

Among 23,821 participants, 2368 (9.9%) individuals were diagnosed with CVD, of which CHD incidence was 1161 (4.9%) and stroke incidence was 1222 (5.1%). In Table 1, compared to patients with constant TC levels before and after T2D diagnosis, patients whose TC levels increased after T2D diagnosis tended to have higher BMI, lower engagement in physical activity, higher blood pressure, higher fasting serum glucose, higher aspartate transaminase (AST), higher alanine transaminase (ALT), higher gamma-GTP, and higher use of lipid-lowering drugs. Among participants on lipid-lowering drugs, over 90% of them in each TC change group used statin.

**Table 1:** Descriptive characteristics of study participants.

TC level before T2D diagnosis (mg/dL)	Low (< 180 mg/dL)			Middle (180–240 mg/dL)			High (≥ 240 mg/dL)		
TC level after T2D diagnosis (mg/dL)	Low	Middle	High	Low	Middle	High	Low	Middle	High
Number of subjects, n (%)	3681 (65.2)	1815 (32.2)	148 (2.6)	4367 (32.9)	7662 (57.7)	1245 (9.4)	1093 (22.3)	2395 (48.8)	1415 (28.9)
Age, years, mean (SD)	61.1 (9.3)	60.5 (9.2)	59.0 (9.1)	59.7 (8.7)	59.3 (8.9)	59.9 (9.1)	59.2 (8.2)	59.4 (8.9)	59.0 (8.7)
Sex, %									
Men	74	65	52	67	63	53	55	56	49
Women	26	35	48	33	37	48	46	44	51
Socioeconomic status, %									
1st quartile (lowest)	16	14	16	15	15	16	14	16	18
2nd quartile	21	23	21	20	20	25	20	21	22
3rd quartile	30	30	34	29	29	30	30	29	32
4th quartile (highest)	35	33	29	36	36	30	36	34	28
BMI, kg/m^2^, mean (SD)	24.5 (3.1)	24.8 (3.1)	25.7 (3.4)	24.9 (3.1)	25.1 (3.1)	25.3 (3.0)	25.1 (3.0)	25.2 (2.9)	25.3 (3.1)
Smoking status, %									
Non-smoker	78	78	10	79	78	83	82	80	80
Smoker	22	22	20	22	22	17	18	20	20
Alcohol consumption, per week, %									
0	55	57	62	56	56	62	60	60	63
< 1	15	16	14	16	16	14	15	14	14
1–2	15	12	12	14	15	12	13	14	11
3–4	9	10	9	10	9	8	8	8	8
≥ 5	6	6	3	5	5	4	4	4	4
Physical activity, per week, %									
0	47	50	58	46	48	53	44	49	51
1–2	23	24	20	24	25	22	25	25	25
3–4	15	13	13	15	14	13	15	13	13
5–6	6	6	4	7	5	5	7	5	4
7	8	8	5	8	8	8	9	8	7
Systolic blood pressure, mmHg, mean (SD)	127.6 (15.8)	129.6(15.6)	130.3 (17.2)	127.5 (15.3)	129.1 (15.5)	130.8 (16.7)	126.5 (14.3)	129.3 (15.4)	131.2 (16.8)
Fasting serum glucose, mg/dL, mean (SD)	126.4 (44.1)	129.8 (44.4)	136.0 (55.7)	122.6 (37.2)	129.0 (42.0)	137.5 (49.9)	121.2 (36.6)	127.8 (40.7)	139.0 (49.1)
Aspartate transaminase, U/L (SD)	30.5 (23.5)	29.7 (17.2)	33.3 (27.6)	28.5 (20.1)	28.3 (17.1)	30.0 (24.6)	28.2 (14.9)	28.4 (15.9)	29.4 (17.2)
Alanine transaminase, U/L (SD)	30.1 (23.1)	31.0 (22.5)	34.3 (20.1)	30.5 (28.6)	30.4 (22.7)	32.5 (28.9)	31.7 (25.5)	30.3 (21.1)	32.1 (26.5)
Gamma-GTP (SD)	54.5 (91.2)	52.5 (68.4)	66.3 (96.5)	46.0 (58.1)	49.5 (63.6)	56.4 (80.2)	48.2 (61.6)	53.5 (70.7)	61.7 (78.3)
Use of lipid-lowering drugs, %									
No	72	76	46	53	70	49	14	43	37
Statin	26	22	53	45	28	50	86	55	62
Others (fibrate, ezetimibe) alone	2	2	1	2	2	1	1	2	1
Anti-diabetic medication, %									
Metformin only	19	18	20	20	17	16	21	15	14
Metformin and sulfonylurea	45	47	45	43	50	52	39	50	52
Other drugs	36	35	36	38	33	32	40	35	33

*TC* total cholesterol, *T2D* type 2 diabetes mellitus, *SD* standard deviation, *n* number, *BMI* body mass index.

In the Kaplan–Meier plot, cumulative probability of non-fatal CVD incidence among T2D diabetic patients during the study follow-up was significantly higher in low–middle and low–high groups compared with low–low group (p = 0.01, Supplementary Fig. 1); in middle–high group compared with middle–middle group (p < 0.001, Supplementary Fig. 2). On the other hand, cumulative probability of CVD incidence among T2D diabetic patients was significantly lower in middle–low group compared with middle–middle group (p < 0.001, Supplementary Fig. 2); in high–low and high–middle groups compared with high–high group (p < 0.0001, Supplementary Fig. 3).

In Table 2, increases in TC levels after T2D diagnosis were generally associated with increased CVD risk, while decreases in TC levels after T2D diagnosis were generally associated with decreased CVD risk. Compared with low–low, aHR of CVD was 1.31 (95% CI 1.10–1.56) for low–middle and 1.80 (95% CI 1.15–2.83) for low–high. Among diabetic patients who were non-users of lipid-lowering drugs, CVD risk increased for low–middle (aHR 1.27, 95% CI 1.04–1.56) and for low–high (aHR 2.25, 95% CI 1.27–3.97). Among diabetic patients who were users of lipid-lowering drugs, CVD risk increased in low–middle (aHR 1.49, 95% CI 1.04–2.14). Compared with middle–middle, aHR of CVD was 1.08 (95% CI 0.82–1.41) for middle–high and 0.75 (95% CI 0.61–0.93) for middle–low. The aHR of CVD in diabetic patients comparing middle–low vs. middle–middle TC levels was 0.86 (95% CI 0.73–1.01) among non-users of lipid-lowering drugs and 0.75 (95% CI 0.61–0.93) among users of lipid-lowering drugs. Compared with high–high, aHR of CVD was 0.65 (95% CI 0.49–0.86) for high–low and 0.68 (95% CI 0.56–0.83) for high–middle. Among diabetic patients who were non-users of lipid-lowering drugs, CVD risk decreased in high–middle (aHR 0.62, 95% CI 0.47–0.82). Among diabetics who were users of lipid-lowering drugs, CVD risk decreased in high–middle (aHR 0.76, 95% CI 0.56–0.99) and high–low (aHR 0.69, 95% CI 0.49–0.96). For all the subgroup analyses by the use of lipid-lowing drugs, there was no evidence of interaction between change in TC levels and use of lipid-lowering drugs (P for interaction > 0.05).

**Table 2:** Associations between change in TC and risk of CVD.

TC level before T2D diagnosis (mg/dL)	Low (< 180 mg/dL)			Middle (180 to 240 mg/dL)			High (≥ 240 mg/dL)		
TC level after T2D diagnosis (mg/dL)	Low	Middle	High	Low	Middle	High	Low	Middle	High
Number of subjects (N)	3681	1815	148	4367	7662	1245	1093	2395	1415
CVD (I20–I25, I60–I69)									
All									
Number of cases (n)	322	212	21	348	799	161	74	230	201
aHR	1	1.31	1.80	0.83	1	1.10	0.65	0.68	1
95% CI	Reference	1.10–1.56	1.15–2.83	0.73–0.94	Reference	0.92–1.31	0.49–0.86	0.56–0.83	Reference
Among non-users of lipid-lowering drugs									
Subtotal (N)	2657	1386	68	2324	5394	605	151	1038	516
Number of cases (n)	245	161	13	206	575	88	18	118	92
aHR	1	1.27	2.25	0.86	1	1.12	0.65	0.62	1
95% CI	Reference	1.04–1.56	1.27–3.97	0.73–1.01	Reference	0.89–1.41	0.39–1.09	0.47–0.82	Reference
Among users of lipid-lowering drugs									
Subtotal (N)	1024	429	80	2043	2268	640	942	1357	899
Number of cases (n)	77	51	8	142	224	73	56	112	109
aHR	1	1.49	1.47	0.75	1	1.08	0.69	0.76	1
95% CI	Reference	1.04–2.14	0.70–3.10	0.61–0.93	Reference	0.82–1.41	0.49–0.96	0.58–0.99	Reference
CHD (I20–I25)									
All									
Number of cases (n)	153	100	10	181	390	82	37	109	99
aHR	1	1.31	1.63	0.85	1	1.15	0.61	0.66	1
95% CI	Reference	1.01–1.69	0.85–3.13	0.71–1.02	Reference	0.90–1.46	0.41–0.90	0.50–0.87	Reference
Among non-users of lipid-lowering drugs									
Subtotal (N)	2657	1386	68	2324	5394	605	151	1038	516
Number of cases (n)	111	67	4	95	268	43	9	46	43
aHR	1	1.14	1.39	0.86	1	1.24	0.70	0.50	1
95% CI	Reference	0.84–1.55	0.51–3.81	0.68–1.08	Reference	0.89–1.71	0.33–1.48	0.33–0.77	Reference
Among users of lipid-lowering drugs									
Subtotal (N)	1024	428	80	2043	2268	640	942	1357	899
Number of cases (n)	42	33	6	86	122	39	28	63	56
aHR	1	1.77	2.08	0.84	1	1.09	0.64	0.83	1
95% CI	Reference	1.11–2.81	0.86–5.01	0.63–1.10	Reference	0.76–1.57	0.40–1.03	0.57–1.19	Reference
Stroke (I60–I69)									
All									
Number of cases (n)	172	114	12	170	413	80	37	122	102
aHR	1	1.32	2.11	0.80	1	1.05	0.69	0.71	1
95% CI	Reference	1.04–1.68	1.16–3.83	0.67–0.96	Reference	0.82–1.34	0.47–1.02	0.54–0.93	Reference
Among non-users of lipid-lowering drugs									
Subtotal (N)	2657	1386	68	2324	5394	605	151	1038	516
Number of cases (n)	135	95	10	114	309	45	9	73	49
aHR	1	1.39	3.03	0.88	1	1.02	0.60	0.74	1
95% CI	Reference	1.07–1.82	1.70–6.40	0.71–1.09	Reference	0.74–1.40	0.29–1.25	0.51–1.07	Reference
Among users of lipid-lowering drugs									
Subtotal (N)	1024	429	80	2043	2268	640	942	1357	899
Number of cases (n)	37	19	2	56	104	35	28	49	53
aHR	1	1.18	0.72	0.65	1	1.07	0.74	0.68	1
95% CI	Reference	0.67–2.09	0.17–3.06	0.47–0.90	Reference	0.73–1.57	0.46–1.18	0.46–1.02	Reference

*aHR*, adjusted hazard ratio analyzed by Cox proportional hazards regression analysis adjusted for age, sex, socioeconomic status, body mass index, smoking status, alcohol consumption, physical activity, blood pressure, fasting serum glucose, anti-diabetic medication, and lipid-lowering medication.

*TC* total cholesterol, *T2D* type 2 diabetes mellitus, *CVD* cardiovascular disease, *CHD* coronary heart disease, *N, n* number, *CI* confidential interval.

For CHD and stroke risk in relation to TC changes, the associations were consistent with the results of CVD in overall diabetic patients, but heterogeneous results emerged in subgroup analysis by use of lipid-lowering drugs (Table 2). For CHD, an increased risk associated with elevated TC was evident in users of lipid-lowering drugs, with aHR comparing low–middle vs. low–low being 1.77 (95% CI 1.11–2.81) in users of lipid-lowering drugs but 1.14 (95% CI 0.84–1.55) in non-users of lipid-lowering drugs. This heterogeneous results by use of lipid-lowering drugs were consistently observed in the results for low–high vs. low–low, albeit not statistically significant due to small number of cases.

In contrast, a decreased risk associated with lowered TC was evident in non-users of lipid-lowering drugs, with aHR comparing high–middle vs. high–high being 0.50 (95% CI 0.33–0.77) in non-users of lipid-lowering drugs but 0.83 (95% CI 0.57–1.19) in users of lipid-lowering drugs.

In contrast, for stroke, an increased risk associated with elevated TC was evident in non-users of lipid-lowering drugs, with aHR comparing low–high vs. low–low being 3.03 (95% CI 1.70–6.40) in non-users of lipid-lowering drugs but 0.72 (95% CI 0.17–3.06) in users of lipid-lowering drugs; but a decreased risk associated with lowered TC in high–middle vs. high–high was suggestive regardless of use of lipid-lowering drugs.

For CVD, CHD, stroke outcomes, additional analyses were performed. In sensitivity analyses conducted among statin users, the results did not change materially compared to the results among users of any lipid-lowering drugs (Supplementary Table 1). In subgroup analyses conducted among participants with information on HDL-C, LDL-C, and TG levels, changes in HDL-C and TG levels after T2D diagnosis were not associated with CVD risk. In contrast, every 10 mg/dL increase in LDL-C levels from pre- to post-diagnosis of T2D was associated with an increased risk of CVD, CHD, which was more pronounced among users of lipid-lowering drugs (aHR 1.02–1.11, 95% CI 1.02–1.11 for CVD; aHR 1.10, 95% CI 1.04–1.17 for CHD) (Supplementary Table 2).

Table 3 presents the results by subtypes of CHD and stroke. While some of the results were statistically unreliable due to a small number of cases, the overall pattern of increasing risk with increasing TC levels and decreasing risk with decreasing TC levels after T2D diagnosis was more evident for angina, MI, and ischemic stroke, all of which are of ischemic origin. For examples, compared with low–low, aHR for low–middle was 1.15 (95% CI 0.84–1.59) for angina, 2.24 (95% CI 1.28–3.91) for MI, 1.38 (95% CI 1.03–1.87) for ischemic stroke; compared with high–high, aHR for high–middle was 0.62 (95% CI 0.45–0.87) for angina, 0.73 (95% CI 0.42–1.28) for MI, 0.60 (95% CI 0.42–0.84) for ischemic stroke.

**Table 3:** Associations between change in TC and risk of CHD and stroke subtypes.

TC level before T2D diagnosis (mg/dL)	Low (< 180 mg/dL)			Middle (180–240 mg/dL)			High (≥ 240 mg/dL)		
TC level after T2D diagnosis (mg/dL)	Low	Middle	High	Low	Middle	High	Low	Middle	High
Number of subjects (N)	3681	1815	148	4367	7663	1245	1093	2395	1415
CHD subtypes									
Angina (I20)									
Number of cases (n)	104	61	7	129	258	55	27	75	72
aHR	1	1.15	1.59	0.92	1	1.15	0.63	0.62	1
95% CI	Reference	0.84–1.59	0.73–3.46	0.74–1.14	Reference	0.85–1.55	0.40–0.99	0.45–0.87	Reference
Chronic IHD (I25)									
Number of cases (n)	37	26	1	41	97	17	9	28	23
aHR	1	1.41	0.66	0.79	1	0.96	0.61	0.73	1
95% CI	Reference	0.85–2.35	0.09–4.85	0.54–1.14	Reference	0.57–1.63	0.28–1.35	0.42–1.28	Reference
Myocardial infarction (I21–I24)									
Number of cases (n)	25	26	2	30	90	16	7	24	18
aHR	1	2.24	2.72	0.63	1	1.07	0.57	0.78	1
95% CI	Reference	1.28–3.91	0.62–11.99	0.41–0.95	Reference	0.62–1.84	0.23–1.41	0.41–1.47	Reference
Stroke subtypes									
Ischemic stroke (I63)									
Number of cases (n)	109	74	8	96	265	51	23	70	69
aHR	1	1.38	2.35	0.72	1	1.05	0.62	0.60	1
95% CI	Reference	1.03–1.87	1.12–4.89	0.57–0.91	Reference	0.77–1.42	0.38–1.01	0.42–0.84	Reference
Other stroke (I67–I69)									
Number of cases (n)	27	27	3	42	69	17	11	28	20
aHR	1	1.93	3.06	1.18	1	1.40	1.00	0.89	1
95% CI	Reference	1.12–3.32	0.89–10.50	0.80–1.74	Reference	0.81–2.40	0.46–2.15	0.49–1.59	Reference
Cerebral infarction without ischemic stroke (I65–I66)									
Number of cases (n)	18	10	2	23	62	13	9	18	13
aHR	1	0.98	3.09	0.69	1	1.13	1.47	0.84	1
95% CI	Reference	0.45–2.14	0.68–13.95	0.42–1.11	Reference	0.62–2.08	0.60–3.56	0.40–1.74	Reference
Hemorrhagic stroke (I60–I62)									
Number of cases (n)	25	15	1	23	52	5	3	14	8
aHR	1	1.16	1.14	0.87	1	0.51	0.71	1.03	1
95% CI	Reference	0.61–2.21	0.15–8.59	0.53–1.43	Reference	0.20–1.29	0.18–2.80	0.42–2.51	Reference

*aHR* adjusted hazard ratio analyzed by Cox proportional hazards regression analysis adjusted for age, sex, socioeconomic status, body mass index, smoking status, alcohol consumption, physical activity, blood pressure, fasting serum glucose, anti-diabetic medication, and lipid-lowering medication.

*TC* total cholesterol, *T2D* type 2 diabetes mellitus, *CHD* coronary heart disease, *N, n* number, *CI* confidential interval, *IHD* ischemic heart disease.

*Results for a stroke subtype (not specified as hemorrhage or infarction stroke, I64) were not provided due to small number of cases (n = 21).

Table 4 shows factors indicative of TC reductions among diabetic patients who were non-users of lipid-lowering drugs. For any of high or middle TC levels before T2D diagnosis, male sex and low fasting glucose levels after T2D diagnosis were associated with approximately 1.34- to 1.78-fold increased odds of TC reduction after T2D diagnosis. On the contrary, Table 5 shows factors indicative of non-improvements in TC levels among diabetic patients who were lipid-lowering drugs users. Overall, female sex, high blood pressure, and high fasting glucose level after T2D diagnosis were suggestive of lipid-lowering drugs resistance, with OR of as non-decreasing or even increasing TC levels ranging from 1.23- to 2.00-fold despite use of lipid-lowering drugs. Supplementary 3 and 4, we performed same analysis of Tables 4 and 5, respectively and lipid-lowering drugs were substituted with statins. The results replaced by statin were similar to those of lipid-lowering drugs.

**Table 4:** Multivariate-adjusted OR of TC decrease after T2D diagnosis among non-users of lipid-lowering drugs.

	Among non-users of lipid-lowering drugs	
	High–middle or high–low vs high–high (ref.)	Middle–low vs middle–middle or middle–high (ref.)
Age, years		
< 60	0.79 (0.63–1.00)	0.97 (0.88–1.08)
≥ 60	1.00 (ref.)	1.00 (ref.)
Sex		
Men	1.78 (1.36–2.31)	1.44 (1.28–1.63)
Women	1.00 (ref.)	1.00 (ref.)
Socioeconomic status		
1st quartile (lowest)	1.00 (ref.)	1.00 (ref.)
2nd quartile	1.00 (0.72–1.38)	0.98 (0.83–1.15)
3rd quartile	1.07 (0.79–1.46)	0.98 (0.84–1.14)
4th quartile (highest)	1.44 (1.05–1.99)	0.92 (0.79–1.07)
BMI, kg/m^2^		
< 25	1.13 (0.91–1.40)	1.17 (1.06–1.29)
≥ 25	1.00 (ref.)	1.00 (ref.)
Smoking status		
Non-smoker	1.07 (0.80–1.44)	1.13 (0.99–1.28)
Smoker	1.00 (ref.)	1.00 (ref.)
Physical activity, per week		
None	1.00 (ref.)	1.00 (ref.)
1–2	0.90 (0.69–1.17)	0.99 (0.87–1.12)
3–4	1.03 (0.72–1.4)	1.03 (0.89–1.20)
5–6	1.01 (0.58–1.76)	1.29 (1.04–1.63)
7	1.04 (0.70–1.53)	1.02 (0.85–1.22)
Alcohol consumption, per week		
No	0.95 (0.74–1.23)	1.09 (0.97–1.22)
Yes	1.00 (ref.)	1.00 (ref.)
Systolic blood pressure, mmHg		
< 120	1.25 (0.94–1.66)	1.22 (1.08–1.38)
120–129.9	1.02 (0.79–1.33)	1.10 (0.97–1.24)
≥ 130	1.00 (ref.)	1.00 (ref.)
Fasting serum glucose, mg/dL		
< 100	1.59 (1.20–2.10)	1.59 (1.40–1.81)
100–125.9	1.77 (1.38–2.27)	1.34 (1.20–1.49)
≥ 126	1.00 (ref.)	1.00 (ref.)

*TC* total cholesterol, *OR* odds ratio.

**Table 5:** Multivariate-adjusted OR of TC increase or non-decrease after T2D diagnosis among users of lipid-lowering drugs.

	Among users of lipid-lowering drugs		
	Low–middle or low–high vs. low–low (ref.)	Middle–middle or middle–high vs. middle–low (ref.)	high–high vs high–middle or high–low (ref.)
Age, years			
< 60	1.00 (ref.)	1.00 (ref.)	1.00 (ref.)
≥ 60	0.76 (0.61–0.95)	0.93 (0.83–1.05)	0.98 (0.83–1.16)
Sex			
Men	1.00 (ref.)	1.00 (ref.)	1.00 (ref.)
Women	1.88 (1.44–2.46)	1.43 (1.24–1.65)	1.32 (1.08–1.60)
Socioeconomic status			
1st quartile (lowest)	1.05 (0.75–1.46)	1.08 (0.90–1.29)	1.28 (1.01–1.64)
2nd quartile	1.01 (0.74–1.37)	1.11 (0.94–1.29)	1.16 (0.93–1.45)
3rd quartile	0.88 (0.67–1.15)	1.02 (0.89–1.18)	1.25 (1.02–1.52)
4th quartile (highest)	1.00 (ref.)	1.00 (ref.)	1.00 (ref.)
BMI, kg/m^2^			
< 25	1.00 (ref.)	1.00 (ref.)	1.00 (ref.)
≥ 25	1.02 (0.83–1.28)	0.90 (0.80–1.01)	0.98 (0.83–1.15)
Smoking status			
Non-smoker	1.00 (ref.)	1.00 (ref.)	1.00 (ref.)
Smoker	1.38 (1.02–1.88)	0.99 (0.84–1.15)	1.34 (1.07–1.68)
Physical activity, per week			
None	1.34 (0.86–2.10)	1.19 (0.94–1.51)	1.50 (1.08–2.09)
1–2	1.21 (0.75–1.94)	1.11 (0.86–1.43)	1.35 (0.95–1.92)
3–4	0.81 (0.49–1.35)	0.99 (0.76–1.30)	1.46(1.00–2.13)
5–6	0.74 (0.40–1.39)	0.77 (0.56–1.05)	0.92 (0.81–1.19)
7	1.00 (ref.)	1.00 (ref.)	1.00 (ref.)
Alcohol consumption, per week			
No	1.00 (ref.)	1.00 (ref.)	1.00 (ref.)
Yes	1.34 (1.03–1.75)	0.92 (0.80–1.05)	0.98 (0.81–1.19)
Systolic blood pressure, mmHg			
< 120	1.00 (ref.)	1.00 (ref.)	1.00 (ref.)
120–129.9	1.15 (0.83–1.59)	1.09 (0.92–1.13)	0.81 (0.64–1.02)
≥ 130	1.53 (1.16–2.01)	1.23 (1.07–1.42)	1.20 (0.99–1.47)
Fasting serum glucose, mg/dL			
< 100	1.00 (ref.)	1.00 (ref.)	1.00 (ref.)
100–125.9	1.11 (0.83–1.49)	1.14 (0.98–1.33)	1.39 (1.10–1.76)
≥ 126	1.46 (1.08–2.00)	1.70 (1.45–1.99)	2.00 (1.59–2.52)

*TC* total cholesterol, *OR* odds ratio.

## Discussion

In patients with T2D, increases in TC level from pre- to post-diagnosis period were associated with elevated CVD risks, while decreases in TC levels were associated with lowered CVD risks. These trends were observed for CVD outcome regardless of use of lipid-lowering drugs and for both CHD and stroke, and more apparent in ischemic diseases than hemorrhagic diseases. Of note, the results for CHD and stroke became heterogeneous when stratified by use of lipid-lowering drugs. In diabetic patients, male sex and low fasting glucose levels were associated with TC reduction without use of lipid-lowering drugs, while female sex, high fasting glucose level, and high blood pressure were associated with non-improvements in TC levels despite use of lipid-lowering drugs.

In generally healthy populations, an elevated cholesterol level in the blood is an established risk factor of CVD^10–13^. Excessive cholesterols, particularly LDL-C, build up in the walls of arteries, forming plaques that narrow or block the arteries that feed the heart or brain^14,15^. Alternatively, the atherosclerotic plaque could be ruptured and the resulting blood clots could travel through vessels and block small vessels that flow to the heart or brain^16,17^. These blockages deprive the heart or brain tissues of blood and oxygen, leading to tissue damage or death^16,18^. Compared to non-diabetic individuals, patients with T2D are at higher risk for hypercholesterolemia, because insulin resistance and ensuing increases in fatty acids flux to the liver lead to an increased secretion of very low density lipoprotein, which converts to LDL in the bloodstream^17^. Increases in insulin levels are also positively correlated with increases in gene expression of 3-hydroxy-3-methylglutaryl-CoA reductase (HMGR), a rate-limiting enzyme of the cholesterol biosynthetic pathway^18^. Furthermore, T2D patients often have smaller LDL particles, which are more atherogenic than normal size LDL^19^. In a previous study conducted among healthy adults aged 20–39 years from this cohort, an increase in TC levels from low to high was associated with 1.2-fold increased CVD risk^10^, which is lower than 1.8-fold increased CVD risk in diabetic patients in our study. Nevertheless, we cannot rule out the possibility that the stronger association observed in our study could be due to the older age of the diabetic patients (59–61 years of age) rather than the interplay of TC increase and diabetes.

With T2D shown to be a major risk factor of CVD, use of lipid-lowering drugs is recommended for diabetic patients ≥ 40 years regardless of their baseline cholesterol levels^20^. One study followed T2D patients to examine the relationship between cholesterol level at T2D diagnosis and the risk of MI and stroke while considering statin use^8^. In this study, among statin users, an increased risk of CVD starts to be observed from LDL-C levels of ≥ 70 mg/dL, while among statin non-users, the LDL-C cut-off for an increased risk of CVD was much higher, of ≥ 130 mg/dL. Of note, our study, which investigated change in TC levels from pre- to post-diagnosis of T2D rather than TC levels at a point in time, performed subgroup analyses by use of lipid-lowering drugs. We observed that a decrease in TC after T2D diagnosis was more evidently associated with a lowered CHD risk among non-users of lipid-lowering drugs, while the decrease was associated with an elevated stroke risk among users of lipid-lowering drugs. Our results suggest that not only changes in TC levels, but also how the changes were induced might influence the disease risk. When cholesterol reduction was achieved through lifestyle modifications alone, because healthy diet and lifestyle affect a broad range of metabolic profiles accompanying LDL reduction, HDL increase, and improved glucose control^21–23^, all of which help reduce CHD risk. In contrast, when cholesterol reduction was achieved via lipid-lowering drugs, its effect is rather specific to LDL reduction^24^ and emerging evidence suggests that lipid-lowering drugs might increase blood glucose levels in pre-diabetic or diabetic people^25^.

Indeed, in our study, non-users of lipid-lowering drugs who managed to reduce TC levels after T2D diagnosis were associated with lower fasting serum glucose levels, whereas users of lipid-lowering drugs who failed to reduce TC levels were associated with higher fasting serum glucose levels. Thus, lipid-lowering drugs’s benefit on cholesterol control might be in part offset by its adverse effect on glucose control. Furthermore, users of lipid-lowering drugs, despite their unhealthy eating habits, might still managed to control their cholesterol levels due to lipid-lowering drugs effect. Taken together, cholesterol improvement itself via use of lipid-lowering drugs might not be strong enough to reduce CHD risk unless other co-risk factors improve and its beneficial effect on stroke might be attributable to lipid-lowering drugs’s other effects. For instance, statin has shown to reduce blood pressure^26^, which appears more protective against stroke than against heart disease^27^. This explanation is consistent with our observation that among users of lipid-lowering drugs, an increased cholesterol level was more evidently associated with an elevated CHD risk than with stroke.

In our analyses by CVD subtypes, associations with TC changes were more evident for advanced ischemic diseases such as angina and MI than chronic IHD, and for ischemic stroke than hemorrhagic stroke. These results are consistent with the mechanism that high cholesterol levels, by forming atherosclerotic plaque and blocking arterial blood vessels, elevates CVD risks. For ischemic vs. hemorrhagic stroke, with ischemic stroke accounting for approximately 80% of all strokes^28^, its more pronounced associations might be in part attributable to statistical power. Of note, the largest proportion of total body cholesterol is contained in the brain^29^ and cholesterol is the major component of myelin membranes^30^. While a low cholesterol concentration in the brain could lead to membrane fragility, making the brain vulnerable to hemorrhagic stroke^31^, the brain cholesterol is controlled by local synthesis and independent of circulating cholesterol levels due to the action of the blood–brain barrier^32^. Thus, an association between cholesterol levels in the blood and hemorrhagic stroke appears biologically less plausible. Nevertheless, in previous studies of generally healthy populations, high TC levels in the blood were associated with an increased ischemic stroke risk, but with a decreased hemorrhagic stroke^33,34^.

We observed sex difference with regard to lipid-lowering drugs’s TC lowering effect. Women were more likely to experience increases or non-decreases in TC levels despite use of lipid-lowering drugs. Such sex disparity was also observed in a study of Taiwanese CHD patients in which women taking stains were less likely to achieve < 160 mg/dL of TC levels compared with men taking equivalent dose of stains^35^. One potential explanation relates to estrogens, which have been suggested to protect women from CVD^36^. CVD is less prevalent in premenopausal women than men and women experience an increased rate of CVD after the onset of menopause, with estrogen replacement therapy resulting in improved blood lipid profiles in postmenopausal women^36^. Although the mechanism underlying cholesterol-lowering effect of estrogen remains elusive, estrogens have been reported to increase cholesterol clearance via increasing LDL receptors and to decrease cholesterol synthesis via inhibiting HMGR^36^. In an experimental study, HMGR activity and expression were lower in female rats and in 17-*β*-estradiol treated male rats than in male rats^37^. Given an already decreased activity HMGR by estrogens in women, stains that inhibit HMGR to reduce LDL are less likely to benefit women than men. Similarly, under the presence of cholesterol-lowering effect of estrogen in women, the beneficial effects of lifestyle modification on cholesterol levels are less likely to manifest in women than in men, which was observed in our study.

Our study has several strengths. To our knowledge, this is the first study that examined changes in TC levels from pre- to post-diagnosis among diabetic patients in relation to subsequent CVD risk. By analyzing changes in TC levels rather than the level at one time point, our study mimics an intervention study on cholesterol levels and disease risk, which better elucidates causality of the relationship.

Yet, several limitations deserve attention. First, inaccuracy inherent in NHIS claims data may compromise the validity of our findings. For instance, previous studies on diagnosis codes found that 70% of the claims data were consistent with patients' medical records^38,39^. To address this limitation, we defined our cohort of diabetic patients based on the combination of ICD-10 codes, hospitalization record, and prescription of anti-diabetic medication. Second, since information on HDL-C and LDL-C was only recently introduced in the NHIS-HEALS database, we could not conduct our study by subtypes of cholesterols. As changes in TC levels could be driven by HDL-C or LDL-C, analysis using TC might have attenuated the true relationships between cholesterol change and CVD risk among diabetic patients. However, because the major benefit of lipid-lowering drugs is lowering LDL-C, our subgroup analysis by among users of lipid-lowering drugs helps understand the effect of LDL-C reduction on CVD risk among diabetic patients. Finally, recruitment period of diabetic patients in our study spans a long period from 2003 up to 2012. Over this time period, the prescription of metformin was increasing while that of sulfonylurea was decreasing in treating diabetic patients^40^ and use of different anti-diabetic medication could have differential effect on cholesterol level and CVD risk. While we adjusted for type of anti-diabetic medication use during two years after T2D diagnosis, residual confounding by change in anti-diabetic medication use over time cannot be completely ruled out.

In conclusion, among diabetic patients, regardless of use of lipid-lowering drugs, increases in TC level from pre- to post-diagnosis period were associated with elevated CVD risks, while the decreases were associated with reduces CVD risks. Management of TC level among diabetic patients may be of an important clinical goal to prevent CVD.

## Methods

### Study population

The National Health Insurance Service (NHIS) in Korea is the mandatory health insurance system that achieved universal coverage of the population since 1989^41^. The NHIS has provided the general health screening programs biennially^42^. To construct the National Health Insurance Service-Health Screening Cohort (NHIS-HEALS), NHIS selected 10% of participants in the 2002–2003 screening program by simple random sampling method. The cohort included 514,866 participants aged 40–79 years in 2002 and followed them through 2015^42^. The cohort had information regarding demographic and socioeconomic factors, medical history, bioclinical laboratory results, and lifestyle factors.

**Figure 1:**
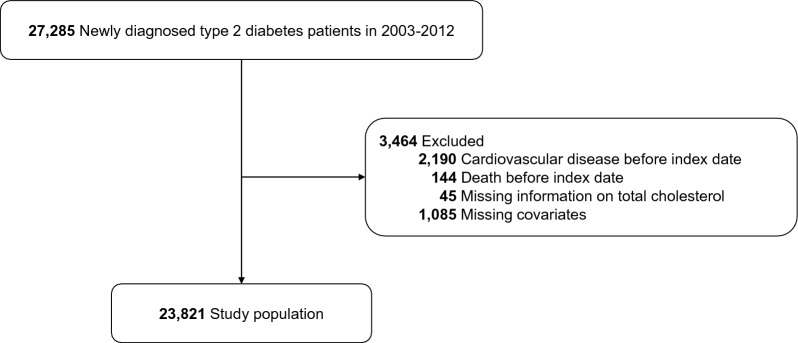
Study population flow.

From the NHIS-HEALS, we selected a total of 27,285 participants who were diagnosed with T2D between 2003 and 2012. Diabetic patients were identified based on International Classification of Diseases 10th Revision (ICD-10) codes (E11, E12, E14) and prescription history of anti-diabetic medication. Among them, we excluded 2334 patients who were diagnosed with CVD or died before the start of study follow-up (i.e., 2 years after T2D diagnosis) and 1130 patients with missing information on TC levels or covariates, leaving 23821 patients for this analysis (Fig. 1).

### Exposure assessment

In the general health examination provided biennially for Koreans aged ≥ 40 years by the NHIS, circulating TC levels were measured via blood test after at least 8 h fasting. Each of TC levels 2 years before and after the diagnosis of T2D was classified into 3 categories: low (< 180 mg/dL), middle (180–239 mg/dL), and high (≥ 240 mg/dL)^10^. Based on these two measurements, changes in TC levels from pre- to post-diagnosis of T2D were divided into 9 groups: low–low, low–middle, low–high, middle–low, middle–middle, middle–high, high–low, high–middle, and high–high.

### Covariable assessment

Covariate information was collected via clinical laboratory test, prescription record, and questionnaire data collected during participants’ visit for the health screening. The time frame was between the date of T2D diagnosis and the date when the follow-up started (i.e., 2 years after T2D diagnosis). Covariates included in the multivariable analysis were as follows: age, sex, socioeconomic status, body mass index (BMI), smoking status, alcohol consumption, physical activity, systolic blood pressure, fasting serum glucose, history of anti-diabetic medication, and use of lipid-lowering drugs after T2D diagnosis. Users of lipid-lowering drugs were defined as those who used all kind of lipid-lowering drugs such as statin, fibrate, ezetimibe, and non-users were as those who did not use any kind of lipid-lowering drugs. Statin users were defined as those who used statin (alone or in combination).

### Outcome ascertainment

The primary outcome was non-fatal CVD incidence, defined as two or more days of hospitalization due to CVD as indicated by ICD-10 codes (I20–I25, I60–I69)^43^. The secondary outcomes were coronary heart disease (CHD, I20–I25) and stroke (I60–I69) incidences. If a patient was diagnosed with both CHD and stoke, the earlier diagnosis was used as the outcome.

### Statistical analysis

**Figure 2:**
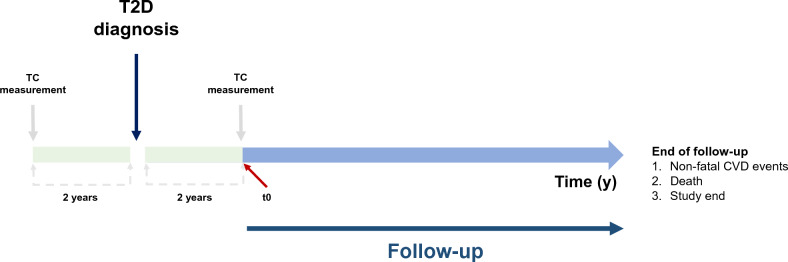
Study design.

For each pre-diagnostic TC category, the reference group included individuals staying in the same category for both pre- and post-diagnostic period (e.g., for low pre-diagnostic TC levels, low–low category was set as the reference against low–middle and low–high categories). For every participant, the start of follow-up (i.e., t0) was set to be “two years after T2D diagnosis”. Defined as “time since t0”, person-years of follow-up were accumulated from this t0 to the date of CVD diagnosis, death, or December 2015, whichever came first (Fig. 2). To estimate cumulative probability of non-fatal CVD incidence according to changes in TC levels from pre- to post-diagnosis of T2D, we used the Kaplan–Meier method and log-rank test. Cox proportional hazards model was used to calculate hazard ratios (HRs) and 95% confidence intervals (CI) of CVD outcomes in relation to change in TC levels from pre- to post-diagnosis of T2D. Heterogeneity in the relationship by CVD subtypes (CHD, stroke) was also explored. For some participants (< 30%) with information on high-density lipoprotein cholesterol (HDL-C), LDL-C and triglycerides (TG), HR and 95% CI of CVD outcomes for a 10 mg/dL increase in HDL-C, LDL-C, and TG levels from pre- to post-diagnosis of T2D were also estimated.

Because lipid abnormalities are common in T2D patients, patients were often prescribed lipid-lowering drugs, which may be an important modifier of the relationship between TC change and CVD risk. Thus, we performed subgroup analysis by any use of lipid-lowering drugsOf note, during our study period, most of the participants on lipid-lowering drugs were prescribed statin alone or in combination with others and only a small proportion (< 10%) used other drugs (e.g., ezetimibe, fibrate) alone^44^. Thus, we also performed sensitivity analysis among statin users. Potential interaction between change in TC levels and use of lipid-lowering drugs was tested by adding their cross-product product term in the model and running the Wald test on it.

Among individuals with high TC levels before T2D diagnosis, some managed to lower their TC levels after T2D diagnosis, which could be attributable to use of lipid-lowering drugs or lifestyle modifications. To identify post-T2D diagnosis factors associated with TC reduction without the help of medication, we performed logistic regression to predict TC decrease (e.g., high–middle or high–low against high–high) using non-medication covariates adjusted in the primary multivariable. On the contrary, among individuals on lipid-lowering drugs, despite their medication use, some failed to lower or even had elevated TC levels after T2D diagnosis. To identify post-T2D diagnosis factors associated with ineffectiveness of lipid lowering medication, we performed logistic regression to predict TC non-decrease or increase (e.g., high–high against high–middle or high–low) using non-medication covariates adjusted for in the primary multivariable analysis.

To explore whether the relationship between TC levels and CVD risk differs by 3rd factors, we performed subgroup analyses by variables selected a priori known to influence CVD risk: age, sex, BMI, smoking status, alcohol consumption, and physical activity (Supplementary table 5).

All statistical analyses were conducted using SAS 9.4 (SAS Institute, NC, USA). Statistically significant results were defined as a two-sided p value less than 0.05.

### Ethical approval

We conducted this study according to the guidelines stipulated in the Declaration of Helsinki. The institutional review board of Seoul National University Hospital approved this study (no. E-2002-040-1099) and informed consent was waived due to the reason that NHIS-HEALS was distributed after being fully anonymized according to strict confidentiality policies.

## Supplementary Information

Supplementary Tables.

Supplementary Figures.

## Data Availability

The database used in this study belongs to the National Health Insurance Service (NHIS), and the authors are not authorized to share the data of this study. The raw NHIS-HEALS database is accessible at https://nhiss.nhis.or.kr/bd/ab/bdaba021eng.do with the permission of the NHIS.
