# Supplementary material for: Changes in total cholesterol level and cardiovascular disease risk among type 2 diabetes patients
Source: Sci Rep. 2023 May 23;13:8342. doi: 10.1038/s41598-023-33743-6 (PMC10205703; doi:10.1038/s41598-023-33743-6)
Supplement: Supplementary file 1 — Supplementary Tables. [file 41598_2023_33743_MOESM1_ESM.docx]

| **Supplementary table 1.** Associations between change in TC and risks of CVD, CHD, and stroke among statin users | | | | | | | | | | |
| --- | --- | --- | --- | --- | --- | --- | --- | --- | --- | --- |
| TC level before T2D diagnosis (mg/dL) | Low (<180 mg/dL) | | | Middle (180 to 240 mg/dL) | | | High (≥240 mg/dL) | | | |
| TC level after T2D diagnosis (mg/dL) | Low | Middle | High | Low | Middle | High | Low | Middle | High |  |
| Number of subjects (N) | 3,681 | 1,815 | 148 | 4,367 | 7,662 | 1,245 | 1,093 | 2,395 | 1,415 |  |
| CVD (I20-I25, I60-I69) |  |  |  |  |  |  |  |  |  |  |
| Among statin users |  |  |  |  |  |  |  |  |  |  |
| Subtotal (N) | 943 | 393 | 78 | 1,970 | 2,127 | 625 | 935 | 1,319 | 883 |  |
| Number of cases (n) | 73 | 49 | 8 | 138 | 212 | 70 | 55 | 106 | 108 |  |
| aHR | 1 | 1.48 | 1.38 | 0.76 | 1 | 1.04 | 0.68 | 0.74 | 1 |  |
| 95% CI | reference | 1.03-2.15 | 0.65-2.91 | 0.61-0.94 | reference | 0.80-1.37 | 0.48-0.95 | 0.56-0.97 | Reference |  |
| CHD (I20-I25) |  |  |  |  |  |  |  |  |  |  |
| Among statin users |  |  |  |  |  |  |  |  |  |  |
| Subtotal (N) | 943 | 393 | 78 | 1,970 | 2,127 | 625 | 935 | 1,319 | 883 |  |
| Number of cases (n) | 42 | 32 | 6 | 82 | 117 | 36 | 28 | 60 | 55 |  |
| aHR | 1 | 1.69 | 1.88 | 0.81 | 1 | 1.01 | 0.65 | 0.81 | 1 |  |
| 95% CI | reference | 1.06-2.70 | 0.78-4.54 | 0.61-1.07 | reference | 0.69-1.46 | 0.41-1.04 | 0.56-1.18 | Reference |  |
| Stroke (I60-I69) |  |  |  |  |  |  |  |  |  |  |
| Among statin users |  |  |  |  |  |  |  |  |  |  |
| Subtotal (N) | 943 | 393 | 78 | 1,970 | 2,127 | 625 | 935 | 1,319 | 883 |  |
| Number of cases (n) | 33 | 18 | 2 | 56 | 97 | 35 | 27 | 46 | 53 |  |
| aHR | 1 | 1.24 | 0.71 | 0.69 | 1 | 1.09 | 0.71 | 0.65 | 1 |  |
| 95% CI | reference | 0.69-2.23 | 0.17-3.05 | 0.49-0.96 | reference | 0.74-1.61 | 0.44-1.15 | 0.44-0.98 | Reference |  |

Abbreviations: TC, total cholesterol; T2D, type 2 diabetes mellitus; CVD, cardiovascular disease; CHD, coronary heart disease; N, n, number; CI, confidential interval

aHR, adjusted hazard ratio analyzed by Cox proportional hazards regression analysis adjusted for age, sex, socioeconomic status, body mass index, smoking status, alcohol consumption, physical activity, blood pressure, fasting serum glucose, anti-diabetic medication, and statin medication

**Supplementary table 2.** Changing of cholesterol levels (10 mg/dl) before and after T2D diagnosis and the risks of CVD, CHD, and stroke

|  | Every 10 mg/dl decrease of  HDL-C change | Every 10 mg/dl increase of  LDL-C change | Every 10 mg/dl increase of  TG change |
| --- | --- | --- | --- |
| CVD (I20-I25, I60-I69) |  |  |  |
| All |  |  |  |
| Number of subtotal/ cases (n) | 6,611 / 238 | 6,221 / 227 | 5,050 / 185 |
| aHR (95% CI) | 0.99 (0.90-1.10) | 1.04 (1.00-1.08) | 1.00 (0.97-1.04) |
| Among non-users of lipid-lowering drugs |  |  |  |
| Number of subtotal, cases (n) | 3,124 / 104 | 3,031 / 102 | 2,472 / 84 |
| aHR (95% CI) | 0.95 (0.81-1.11) | 0.99 (0.93-1.06) | 1.00 (0.95-1.05) |
| Among users of lipid-lowering drugs |  |  |  |
| Number of subtotal, cases (n) | 3,487 / 134 | 3,190 / 125 | 2,477 / 101 |
| aHR (95% CI) | 1.01 (0.89-1.16) | 1.06 (1.02-1.11) | 1.01 (0.97-1.05) |
| CHD (I20-I25) |  |  |  |
| All |  |  |  |
| Number of subtotal/ cases (n) | 6,611 / 116 | 6,221 / 112 | 5,050 / 87 |
| aHR (95% CI) | 0.90 (0.86-1.15) | 1.05 (1.00-1.11) | 1.02 (0.98-1.07) |
| Among non-users of lipid-lowering drugs |  |  |  |
| Number of subtotal/ cases (n) | 3,124 / 48 | 3,031 / 47 | 2,472 / 38 |
| aHR (95% CI) | 0.93 (0.74-1.17) | 0.96 (0.87-1.05) | 1.02 (0.96-1.10) |
| Among users of lipid-lowering drugs |  |  |  |
| Number of subtotal/ cases (n) | 3,487 / 68 | 3,190 / 65 | 2,578 / 49 |
| aHR (95% CI) | 1.03 (0.85-1.25) | 1.10 (1.04-1.17) | 1.02 (0.96-1.08) |
| Stroke (I60-I69) |  |  |  |
| All |  |  |  |
| Number of subtotal/ cases (n) | 6,611 / 125 | 6,221 / 118 | 5,050 / 100 |
| aHR (95% CI) | 0.99 (0.86-1.14) | 1.03 (0.98-1.09) | 0.99 (0.95-1.04) |
| Among non-users of lipid-lowering drugs |  |  |  |
| Number of subtotal/ cases (n) | 3,124 / 57 | 3,031 / 56 | 2,472 / 46 |
| aHR (95% CI) | 0.95 (0.77-1.18) | 1.04 (0.96-1.14) | 0.97 (0.91-1.04) |
| Among users of lipid-lowering drugs |  |  |  |
| Number of subtotal/ cases (n) | 3,487 / 68 | 3,190 / 62 | 2,578 / 54 |
| aHR (95% CI) | 1.01 (0.94-1.12) | 1.03 (0.97-1.10) | 1.00 (0.95-1.06) |

Abbreviations: HDL-C, High-density lipoprotein cholesterol; LDL-C, Low-density lipoprotein cholesterol; TG, triglyceride; CVD, cardiovascular disease; CHD, coronary heart disease; n, number; aHR, adjusted hazard ratio analyzed by Cox proportional hazards regression analysis adjusted for age, sex, socioeconomic status, body mass index, smoking status, alcohol consumption, physical activity, blood pressure, fasting serum glucose, anti-diabetic medication, and lipid-lowering medication

| **Supplementary table 3.** Multivariate-adjusted OR of TC decrease after T2D diagnosis among statin non-users   \|  \| Among non-users of statin \| \| \| --- \| --- \| --- \| \|  \| High-Middle or High-Low vs.  High-High (ref.) \| Middle-Low vs.  Middle-Middle or Middle-High (ref.) \| \| Age, years \|  \|  \| \| <60 \| 0.81 (0.65-1.02) \| 0.98 (0.88-1.08) \| \| ≥60 \| 1.00 (ref.) \| 1.00 (ref.) \| \| Sex \|  \|  \| \| Men \| 1.74 (1.34-2.25) \| 1.44 (1.28-1.62) \| \| Women \| 1.00 (ref.) \| 1.00 (ref.) \| \| Socioeconomic status \|  \|  \| \| 1^st^ quartile (lowest) \| 1.00 (ref.) \| 1.00 (ref.) \| \| 2^nd^ quartile \| 0.98 (0.71-1.34) \| 0.98 (0.83-1.15) \| \| 3^rd^ quartile \| 1.04 (0.76-1.41) \| 0.99 (0.85-1.15) \| \| 4^th^ quartile (highest) \| 1.37 (1.00-1.87) \| 0.92 (0.80-1.07) \| \| BMI, kg/m^2^ \|  \|  \| \| <25 \| 1.14 (0.92-1.41) \| 1.16 (1.06-1.28) \| \| ≥25 \| 1.00 (ref.) \| 1.00 (ref.) \| \| Smoking status \|  \|  \| \| Non-smoker \| 1.12 (0.84-1.48) \| 1.13 (1.01-1.28) \| \| Smoker \| 1.00 (ref.) \| 1.00 (ref.) \| \| Physical activity, per week \|  \|  \| \| None \| 1.00 (ref.) \| 1.00 (ref.) \| \| 1-2 \| 0.91 (0.70-1.18) \| 0.99 (0.87-1.12) \| \| 3-4 \| 1.03 (0.73-1.45) \| 1.03 (0.88-1.19) \| \| 5-6 \| 1.06 (0.61-1.83) \| 1.26 (1.02-1.55) \| \| 7 \| 1.06 (0.72-1.56) \| 1.02 (0.85-1.22) \| \| Alcohol consumption, per week \|  \|  \| \| No \| 0.92 (0.71-1.18) \| 1.07 (0.96-1.20) \| \| Yes \| 1.00 (ref.) \| 1.00 (ref.) \| \| Systolic blood pressure, mmHg \|  \|  \| \| <120 \| 1.26 (0.96-1.67) \| 1.21 (1.07-1.36) \| \| 120-129.9 \| 1.03 (0.80-1.33) \| 1.12 (1.00-1.26) \| \| ≥130 \| 1.00 (ref.) \| 1.00 (ref.) \| \| Fasting serum glucose, mg/dL \|  \|  \| \| <100 \| 1.65 (1.25-2.18) \| 1.62 (1.43-1.84) \| \| 100-125.9 \| 1.81 (1.41-2.31) \| 1.37 (1.23-1.53) \| \| ≥126 \| 1.00 (ref.) \| 1.00 (ref.) \|   Abbreviations: TC, total cholesterol; OR, odds ratio.  **Supplementary Table 4.** Multivariate-adjusted OR of TC increase or non-decrease after T2D diagnosis among statin users   \|  \| Among users of statin \| \| \| \| --- \| --- \| --- \| --- \| \|  \| Low-Middle or Low-High vs. Low-Low (ref.) \| Middle-Middle or Middle-High vs. Middle-Low (ref.) \| High-High vs.  High-Middle or High-Low (ref.) \| \| Age, years \|  \|  \|  \| \| <60 \| 1.00 (ref.) \| 1.00 (ref.) \| 1.00 (ref.) \| \| ≥60 \| 0.76 (0.60-0.96) \| 0.93 (0.83-1.06) \| 0.97 (0.82-1.15) \| \| Sex \|  \|  \|  \| \| Men \| 1.00 (ref.) \| 1.00 (ref.) \| 1.00 (ref.) \| \| Women \| 1.66 (1.26-2.19) \| 1.44 (1.24-1.66) \| 1.32 (1.08-1.61) \| \| Socioeconomic status \|  \|  \|  \| \| 1^st^ quartile (lowest) \| 1.04 (0.74-1.47) \| 1.07 (0.89-1.29) \| 1.32 (1.03-1.68) \| \| 2^nd^ quartile \| 0.99 (0.82-1.36) \| 1.12 (0.95-1.32) \| 1.17 (0.93-1.46) \| \| 3^rd^ quartile \| 0.87 (0.66-1.16) \| 1.04 (0.90-1.20) \| 1.25 (1.02-1.53) \| \| 4^th^ quartile (highest) \| 1.00 (ref.) \| 1.00 (ref.) \| 1.00 (ref.) \| \| BMI, kg/m^2^ \|  \|  \|  \| \| <25 \| 1.00 (ref.) \| 1.00 (ref.) \| 1.00 (ref.) \| \| ≥25 \| 1.02 (0.82-1.29) \| 0.89 (0.79-1.00) \| 0.97 (0.83-1.14) \| \| Smoking status \|  \|  \|  \| \| Non-smoker \| 1.00 (ref.) \| 1.00 (ref.) \| 1.00 (ref.) \| \| Smoker \| 1.36 (0.99-1.88) \| 0.96 (0.81-1.13) \| 1.32 (1.05-1.65) \| \| Physical activity, per week \|  \|  \|  \| \| None \| 1.34 (0.85-2.12) \| 1.19 (0.93-1.52) \| 1.49 (1.07-2.08) \| \| 1-2 \| 1.25 (0.77-2.04) \| 1.10 (0.85-1.43) \| 1.34 (0.94-1.92) \| \| 3-4 \| 0.81 (0.48-1.38) \| 0.99 (0.76-1.30) \| 1.46 (0.99-2.13) \| \| 5-6 \| 0.83 (0.43-1.60) \| 0.73 (0.53-1.01) \| 0.92 (0.57-1.50) \| \| 7 \| 1.00 (ref.) \| 1.00 (ref.) \| 1.00 (ref.) \| \| Alcohol consumption, per week \|  \|  \|  \| \| No \| 1.00 (ref.) \| 1.00 (ref.) \| 1.00 (ref.) \| \| Yes \| 1.25 (0.95-1.65) \| 0.93 (0.81-1.06) \| 1.00 (0.82-1.22) \| \| Systolic blood pressure, mmHg \|  \|  \|  \| \| <120 \| 1.00 (ref.) \| 1.00 (ref.) \| 1.00 (ref.) \| \| 120-129.9 \| 1.19 (0.85-1.53) \| 1.13 (0.96-1.33) \| 0.80 (0.63-1.01) \| \| ≥130 \| 1.54 (1.16-2.05) \| 1.24 (1.07-1.43) \| 1.20 (0.98-1.46) \| \| Fasting serum glucose, mg/dL \|  \|  \|  \| \| <100 \| 1.00 (ref.) \| 1.00 (ref.) \| 1.00 (ref.) \| \| 100-125.9 \| 1.13 (0.84-1.53) \| 1.14 (0.98-1.14) \| 1.38 (1.09-1.75) \| \| ≥126 \| 1.43 (1.05-1.95) \| 1.66 (1.41-1.96) \| 1.97 (1.56-2.48) \|   Abbreviations: TC, total cholesterol; OR, odds ratio. |
| --- | --- | --- | --- | --- | --- | --- | --- | --- | --- | --- | --- | --- | --- | --- | --- | --- | --- | --- | --- | --- | --- | --- | --- | --- | --- | --- | --- | --- | --- | --- | --- | --- | --- | --- | --- | --- | --- | --- | --- | --- | --- | --- | --- | --- | --- | --- | --- | --- | --- | --- | --- | --- | --- | --- | --- | --- | --- | --- | --- | --- | --- | --- | --- | --- | --- | --- | --- | --- | --- | --- | --- | --- | --- | --- | --- | --- | --- | --- | --- | --- | --- | --- | --- | --- | --- | --- | --- | --- | --- | --- | --- | --- | --- | --- | --- | --- | --- | --- | --- | --- | --- | --- | --- | --- | --- | --- | --- | --- | --- | --- | --- | --- | --- | --- | --- | --- | --- | --- | --- | --- | --- | --- | --- | --- | --- | --- | --- | --- | --- | --- | --- | --- | --- | --- | --- | --- | --- | --- | --- | --- | --- | --- | --- | --- | --- | --- | --- | --- | --- | --- | --- | --- | --- | --- | --- | --- | --- | --- | --- | --- | --- | --- | --- | --- | --- | --- | --- | --- | --- | --- | --- | --- | --- | --- | --- | --- | --- | --- | --- | --- | --- | --- | --- | --- | --- | --- | --- | --- | --- | --- | --- | --- | --- | --- | --- | --- | --- | --- | --- | --- | --- | --- | --- | --- | --- | --- | --- | --- | --- | --- | --- | --- | --- | --- | --- | --- | --- | --- | --- | --- | --- | --- | --- | --- | --- | --- | --- | --- | --- | --- | --- | --- | --- | --- | --- | --- | --- | --- | --- | --- | --- | --- | --- | --- | --- | --- | --- | --- | --- | --- | --- | --- |
|  |

| **Supplementary Table 5.** Subgroup analysis of the associations between change in total cholesterol and risk of CVD | | | | | | | | | |
| --- | --- | --- | --- | --- | --- | --- | --- | --- | --- |
| Total cholesterol level before T2D diagnosis (mg/dL) | Low (<180 mg/dL) | | | Middle (180 to 240 mg/dL) | | | High (≥240 mg/dL) | | |
| Total cholesterol level after T2D diagnosis (mg/dL) | Low | Middle | High | Low | Middle | High | Low | Middle | High |
| Age |  |  |  |  |  |  |  |  |  |
| Age<60 |  |  |  |  |  |  |  |  |  |
| Subtotal (N) | 1,730 | 914 | 82 | 2,340 | 4,215 | 640 | 633 | 1,316 | 810 |
| Number of cases (n) | 90 | 69 | 9 | 131 | 316 | 60 | 31 | 93 | 89 |
| aHR | 1 | 1.46 | 1.97 | 0.84 | 1 | 1.10 | 0.64 | 0.66 | 1 |
| 95% CI | reference | 1.06-2.01 | 0.97-3.99 | 0.68-1.04 | reference | 0.84-1.47 | 0.42-0.98 | 0.49-0.89 | reference |
| Age≥60 |  |  |  |  |  |  |  |  |  |
| Subtotal (N) | 1,951 | 901 | 66 | 2,027 | 3,448 | 605 | 460 | 1,079 | 605 |
| Number of cases (n) | 232 | 143 | 12 | 217 | 483 | 101 | 43 | 137 | 112 |
| aHR | 1 | 1.26 | 1.47 | 0.82 | 1 | 1.11 | 0.66 | 0.68 | 1 |
| 95% CI | reference | 1.02-1.56 | 0.81-2.66 | 0.69-0.96 | reference | 0.89-1.38 | 0.46-0.94 | 0.52-0.88 | reference |
| Sex |  |  |  |  |  |  |  |  |  |
| Men |  |  |  |  |  |  |  |  |  |
| Subtotal (N) | 2,726 | 1,181 | 77 | 2,928 | 4,838 | 654 | 596 | 1,348 | 696 |
| Number of cases (n) | 247 | 148 | 10 | 250 | 524 | 82 | 41 | 146 | 96 |
| aHR | 1 | 1.35 | 1.50 | 0.83 | 1 | 1.00 | 0.67 | 0.77 | 1 |
| 95% CI | reference | 1.10-1.66 | 0.79-2.86 | 0.71-0.97 | reference | 0.79-1.27 | 0.46-0.98 | 0.59-1.01 | reference |
| Women |  |  |  |  |  |  |  |  |  |
| Subtotal (N) | 955 | 634 | 71 | 1,439 | 2,825 | 591 | 497 | 1,047 | 719 |
| Number of cases (n) | 75 | 64 | 11 | 98 | 275 | 79 | 33 | 84 | 105 |
| aHR | 1 | 1.23 | 2.54 | 0.80 | 1 | 1.22 | 0.64 | 0.60 | 1 |
| 95% CI | reference | 0.88-1.72 | 1.29-4.97 | 0.64-1.01 | reference | 0.94-1.57 | 0.43-0.97 | 0.44-0.80 | reference |
| BMI |  |  |  |  |  |  |  |  |  |
| BMI<25 |  |  |  |  |  |  |  |  |  |
| Subtotal (N) | 2,154 | 999 | 68 | 2,276 | 3,951 | 594 | 549 | 1,194 | 679 |
| Number of cases (n) | 207 | 125 | 12 | 176 | 414 | 82 | 33 | 117 | 87 |
| aHR | 1 | 1.27 | 2.17 | 0.82 | 1 | 1.14 | 0.60 | 0.70 | 1 |
| 95% CI | reference | 1.02-1.59 | 1.20-3.93 | 0.69-0.99 | reference | 0.90-1.45 | 0.40-0.90 | 0.53-0.93 | reference |
| BMI≥25 |  |  |  |  |  |  |  |  |  |
| Subtotal (N) | 1,527 | 816 | 80 | 2,091 | 3,712 | 651 | 544 | 1,201 | 736 |
| Number of cases (n) | 115 | 87 | 9 | 172 | 385 | 79 | 41 | 113 | 114 |
| aHR | 1 | 1.38 | 1.42 | 0.82 | 1 | 1.04 | 0.70 | 0.65 | 1 |
| 95% CI | reference | 1.04-1.83 | 0.71-2.85 | 0.68-0.99 | reference | 0.81-1.33 | 0.48-1.02 | 0.50-0.85 | reference |
| Smoking status |  |  |  |  |  |  |  |  |  |
| Non-smoker |  |  |  |  |  |  |  |  |  |
| Subtotal (N) | 2,874 | 1,418 | 118 | 3,430 | 6,010 | 1,032 | 894 | 1,923 | 1,128 |
| Number of cases (n) | 235 | 156 | 18 | 281 | 595 | 133 | 57 | 177 | 157 |
| aHR | 1 | 1.26 | 1.89 | 0.91 | 1 | 1.16 | 0.66 | 0.69 | 1 |
| 95% CI | reference | 1.00-1.59 | 1.10-3.24 | 0.78-1.06 | reference | 0.95-1.43 | 0.47-0.93 | 0.55-0.88 | reference |
| Smoker |  |  |  |  |  |  |  |  |  |
| Subtotal (N) | 807 | 397 | 30 | 937 | 1,653 | 213 | 199 | 472 | 287 |
| Number of cases (n) | 87 | 56 | 3 | 67 | 204 | 28 | 17 | 53 | 44 |
| aHR | 1 | 1.33 | 1.29 | 0.63 | 1 | 0.97 | 0.78 | 0.83 | 1 |
| 95% CI | reference | 0.95-1.88 | 0.39-4.35 | 0.47-0.83 | reference | 0.65-1.45 | 0.43-1.40 | 0.54-1.28 | reference |
| Alcohol consumption |  |  |  |  |  |  |  |  |  |
| No |  |  |  |  |  |  |  |  |  |
| Subtotal (N) | 2,035 | 1,029 | 92 | 2,426 | 4,319 | 776 | 657 | 1,440 | 884 |
| Number of cases (n) | 180 | 135 | 14 | 215 | 493 | 112 | 46 | 157 | 138 |
| aHR | 1 | 1.43 | 1.78 | 0.83 | 1 | 1.11 | 0.62 | 0.74 | 1 |
| 95% CI | reference | 1.14-1.80 | 1.02-3.09 | 0.70-0.97 | reference | 0.90-1.37 | 0.44-0.88 | 0.59-0.94 | reference |
| Yes |  |  |  |  |  |  |  |  |  |
| Subtotal (N) | 1,941 | 3,344 | 469 | 1,941 | 3,344 | 469 | 436 | 955 | 531 |
| Number of cases (n) | 133 | 306 | 49 | 133 | 306 | 49 | 28 | 73 | 63 |
| aHR | 1 | 1.09 | 1.54 | 0.83 | 1 | 1.03 | 0.71 | 0.57 | 1 |
| 95% CI | reference | 0.82-1.44 | 0.71-3.36 | 0.67-1.02 | reference | 0.75-1.40 | 0.45-1.13 | 0.40-0.81 | reference |
| Physical activity |  |  |  |  |  |  |  |  |  |
| No |  |  |  |  |  |  |  |  |  |
| Subtotal (N) | 1,723 | 903 | 86 | 1,999 | 3,697 | 665 | 481 | 1,176 | 724 |
| Number of cases (n) | 170 | 115 | 12 | 180 | 424 | 106 | 35 | 115 | 119 |
| aHR | 1 | 1.31 | 1.82 | 0.81 | 1 | 1.23 | 0.56 | 0.58 | 1 |
| 95% CI | reference | 1.03-1.67 | 1.00-3.32 | 0.68-0.97 | reference | 0.99-1.52 | 0.38-0.82 | 0.45-0.76 | reference |
| Yes |  |  |  |  |  |  |  |  |  |
| Subtotal (N) | 1,958 | 912 | 62 | 2,368 | 3,966 | 580 | 612 | 1,219 | 691 |
| Number of cases (n) | 152 | 97 | 9 | 168 | 375 | 55 | 39 | 115 | 82 |
| aHR | 1 | 1.30 | 1.72 | 0.85 | 1 | 0.91 | 0.81 | 0.83 | 1 |
| 95% CI | reference | 1.01-1.68 | 0.87-3.43 | 0.70-1.02 | reference | 0.68-1.22 | 0.54-1.20 | 0.62-1.11 | reference |
| Abbreviations: T2D, type 2 diabetes mellitus; CVD, cardiovascular disease; N, n, number; CI, confidential interval; aHR, adjusted hazard ratio analyzed by Cox proportional hazards regression analysis adjusted for age, sex, body mass index, smoking status, alcohol consumption, physical activity, blood pressure, fasting serum glucose, anti-diabetic medication, and statin medication | | | | | | | | | |
