# Supplementary material for: Changes in total cholesterol level and cardiovascular disease risk among type 2 diabetes patients
Source: Sci Rep. 2023 May 23;13:8342. doi: 10.1038/s41598-023-33743-6 (PMC10205703; doi:10.1038/s41598-023-33743-6)
Supplement: Supplementary file 2 — Supplementary Figures. [file 41598_2023_33743_MOESM2_ESM.pdf]

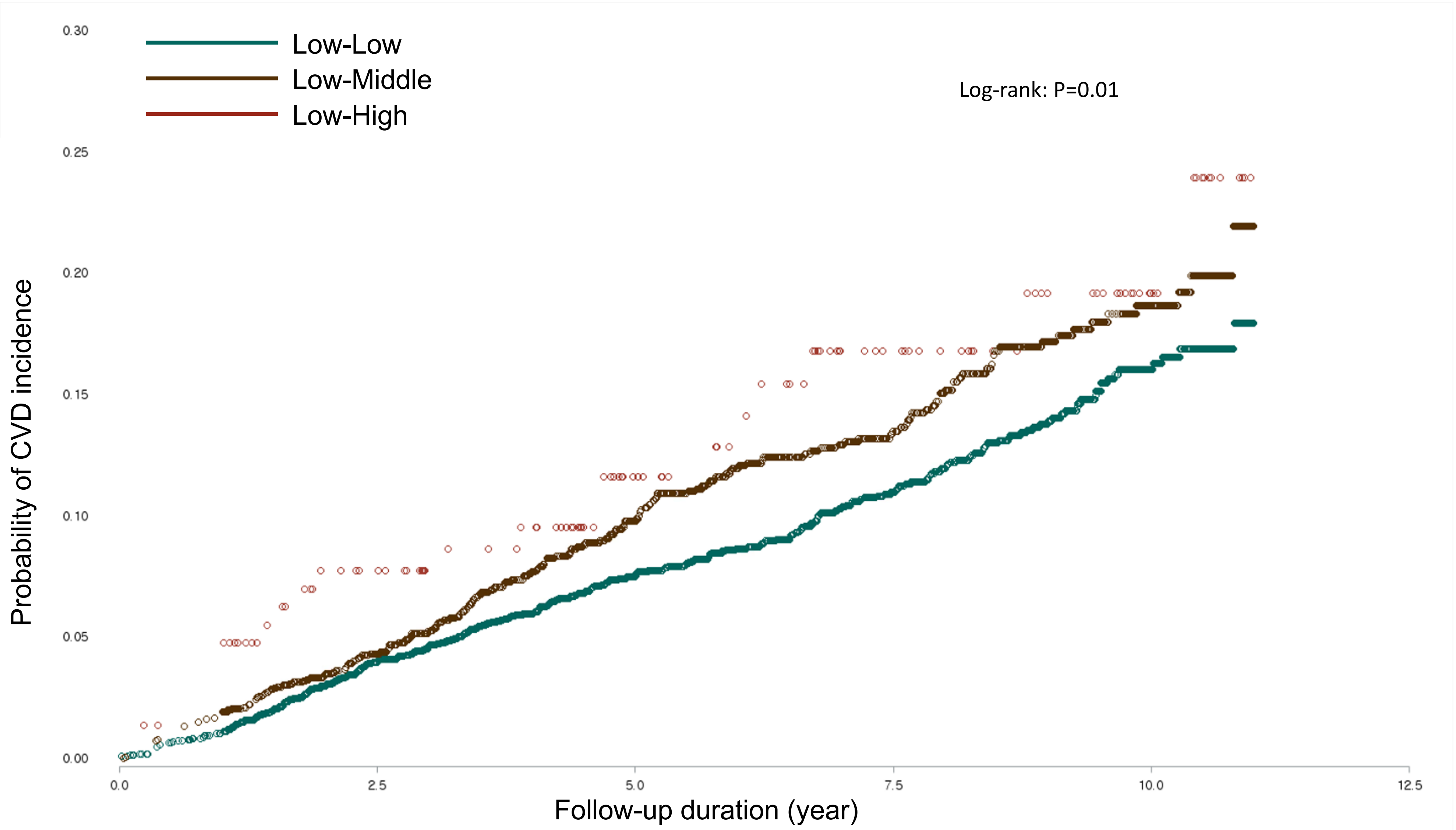

Figure Legend:

Supplementary Figure 1. Kaplan-Meier Estimates of Cumulative Probability of CVD incidence among patients with low cholesterol levels before T2D diagnosis

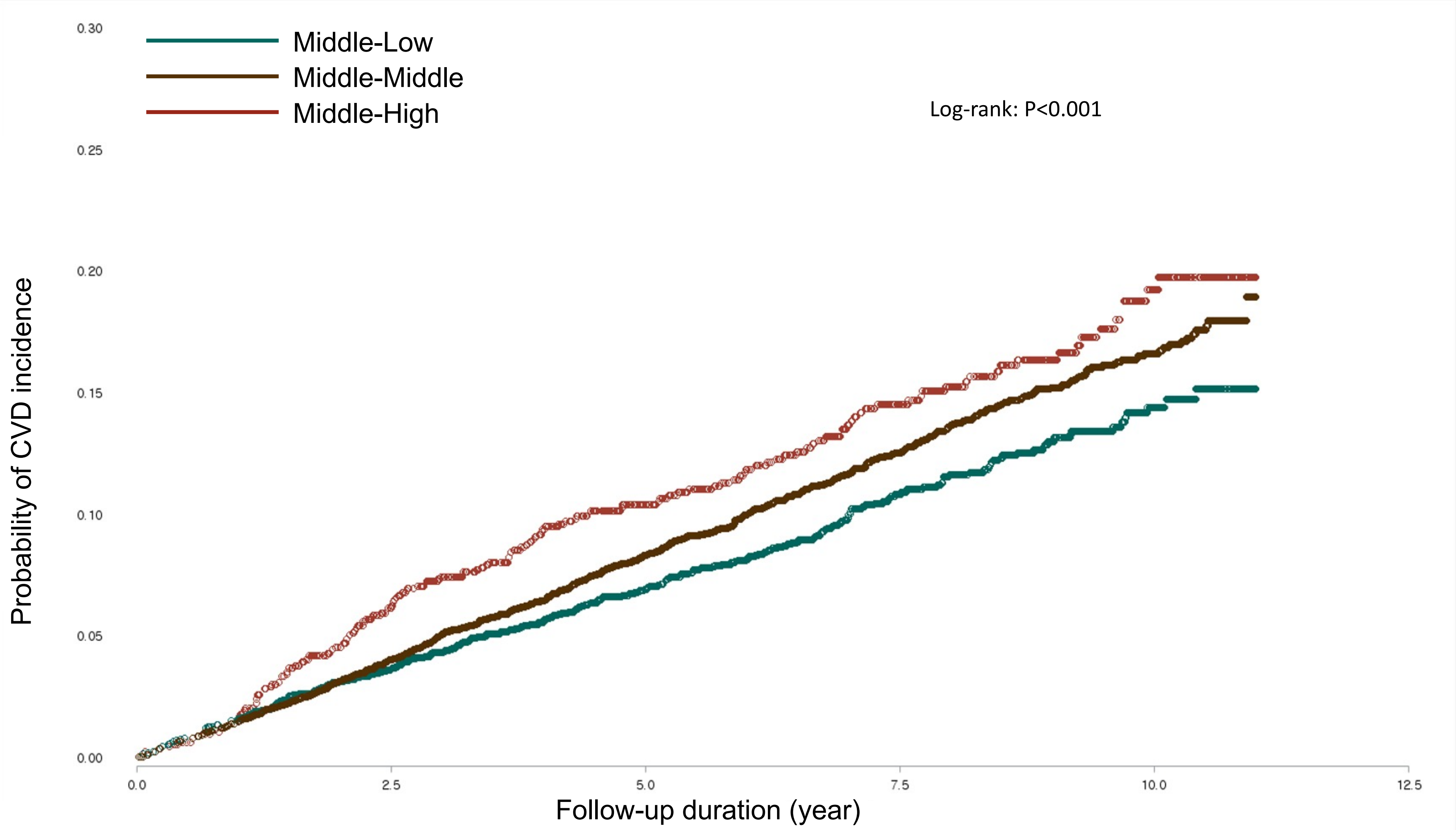

Figure Legend:

Supplementary Figure 2. Kaplan-Meier Estimates of Cumulative Probability of CVD incidence among patients with middle cholesterol levels before T2D diagnosis

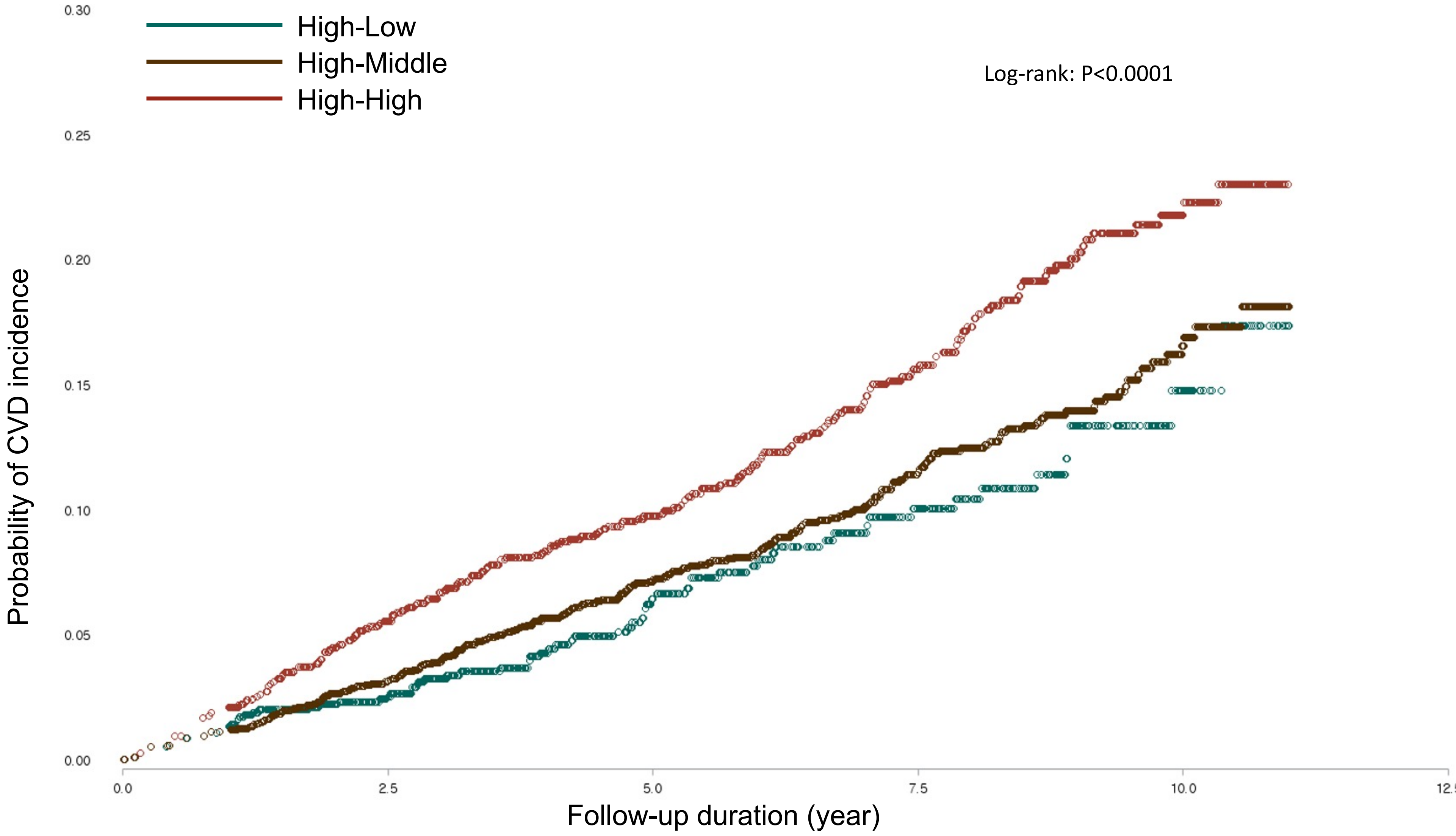

Figure Legend:
Supplementary Figure 3. Kaplan-Meier Estimates of Cumulative Probability of CVD incidence among patients with high cholesterol levels before T2D diagnosis
